# Bis{2-hydr­oxy-*N*-[2-(2-pyrid­yl)eth­yl]benzamide}copper(I) tetra­fluoridoborate

**DOI:** 10.1107/S1600536810001364

**Published:** 2010-01-16

**Authors:** Zhaodong Wang, Douglas R. Powell, Robert P. Houser

**Affiliations:** a Department of Chemistry and Biochemistry, University of Oklahoma, Norman, OK 73019-3051, USA

## Abstract

The title complex, [Cu(C_14_H_14_N_2_O_2_)_2_]BF_4_, is a monomeric copper(I) species with linear two-coordinate geometry around the Cu^I^ atom. The asymmetric unit contains two half-cations that sit on crystallographic twofold rotation axes. The selected crystal was non-merohedrally twinned by a twofold rotation about an axis normal to the (100) family of planes. The ratio of the twin components refined to 0.4123 (6). Two 2-hydr­oxy-*N*-[2-(2-pyrid­yl)eth­yl]benzamide ligands coordinate to each Cu^I^ atom *via* the pyridyl N atom. Intra­molecular hydrogen bonding between the phenol OH groups and the amide O atoms imparts rigidity and planarity to the non-coordinating end of the ligand. The cationic complex is linked to the BF_4_^−^ anions *via* hydrogen bonding between the amide NH groups in the cations and BF_4_^−^ anions.

## Related literature

For the synthesis and coordination chemistry of 2-hydr­oxy-*N*-(2-(2-pyrid­yl)eth­yl)benzamide, see: Wang *et al.* (2009 ▶). For the copper(I) coordination chemistry of pyridylamides, see Yang *et al.* (2007 ▶) and references therein.
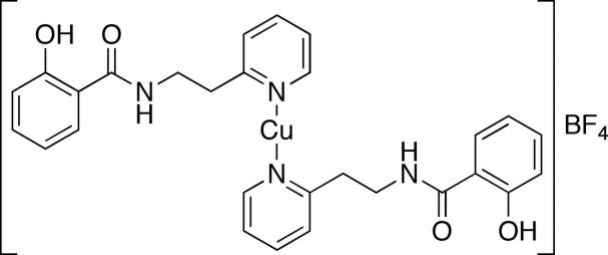


## Experimental

### 

#### Crystal data

[Cu(C_14_H_14_N_2_O_2_)_2_]BF_4_
*M* *_r_* = 634.89
Monoclinic, 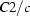

*a* = 21.943 (5) Å
*b* = 17.586 (4) Å
*c* = 14.607 (2) Å
β = 107.988 (8)°
*V* = 5361.2 (19) Å^3^
*Z* = 8
Mo *K*α radiation
μ = 0.89 mm^−1^
*T* = 100 K
0.40 × 0.30 × 0.12 mm

#### Data collection

Bruker APEX CCD diffractometer
Absorption correction: multi-scan (*TWINABS*; Bruker, 2001 ▶) *T*_min_ = 0.714, *T*_max_ = 0.905
10344 measured reflections
10344 independent reflections
8548 reflections with *I* > 2σ(*I*)

#### Refinement

*R*[*F* ^2^ > 2σ(*F* ^2^)] = 0.034
*wR*(*F* ^2^) = 0.096
*S* = 1.00
10344 reflections
393 parameters
4 restraints
H atoms treated by a mixture of independent and constrained refinement
Δρ_max_ = 0.54 e Å^−3^
Δρ_min_ = −0.39 e Å^−3^

### 

Data collection: *SMART* (Bruker, 2007 ▶); cell refinement: *SAINT* (Bruker, 2007 ▶); data reduction: *SAINT*; program(s) used to solve structure: *SHELXTL* (Sheldrick, 2008 ▶); program(s) used to refine structure: *SHELXTL*; molecular graphics: *SHELXTL*; software used to prepare material for publication: *SHELXTL*.

## Supplementary Material

Crystal structure: contains datablocks I, global. DOI: 10.1107/S1600536810001364/bt5166sup1.cif

Structure factors: contains datablocks I. DOI: 10.1107/S1600536810001364/bt5166Isup2.hkl

Additional supplementary materials:  crystallographic information; 3D view; checkCIF report

## Figures and Tables

**Table:** 

Cu1*A*—N1*A*	1.8872 (16)
Cu1*B*—N1*B*	1.8874 (17)

**Table:** 

N1*A*—Cu1*A*—N1*A*^i^	178.45 (9)
N1*B*—Cu1*B*—N1*B*^ii^	177.71 (9)

Symmetry codes: (i) 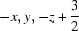; (ii) 
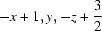
.

**Table 2:** Hydrogen-bond geometry (Å, °)

*D*—H⋯*A*	*D*—H	H⋯*A*	*D*⋯*A*	*D*—H⋯*A*
N9*A*—H9*A*⋯F1^iii^	0.72 (2)	2.23 (2)	2.923 (2)	161 (2)
O18*A*—H18*A*⋯O11*A*	0.83 (1)	1.79 (2)	2.554 (2)	152 (3)
N9*B*—H9*B*⋯F2^iv^	0.82 (1)	2.16 (1)	2.937 (2)	159 (2)
O18*B*—H18*B*⋯O11*B*	0.81 (1)	1.81 (2)	2.549 (2)	153 (2)

Symmetry codes: (iii) 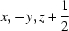; (iv) 
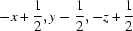
.

**Fig. 1.**
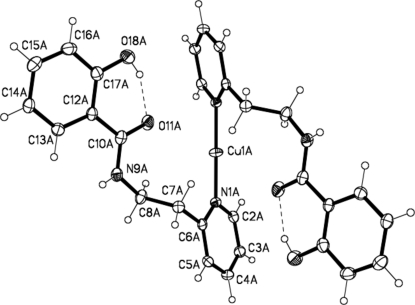
The molecular structure of the cation (molecule A) in [Cu(C14H14N2O2)2]BF4, showing the atom-numbering scheme.

**Fig. 2.**
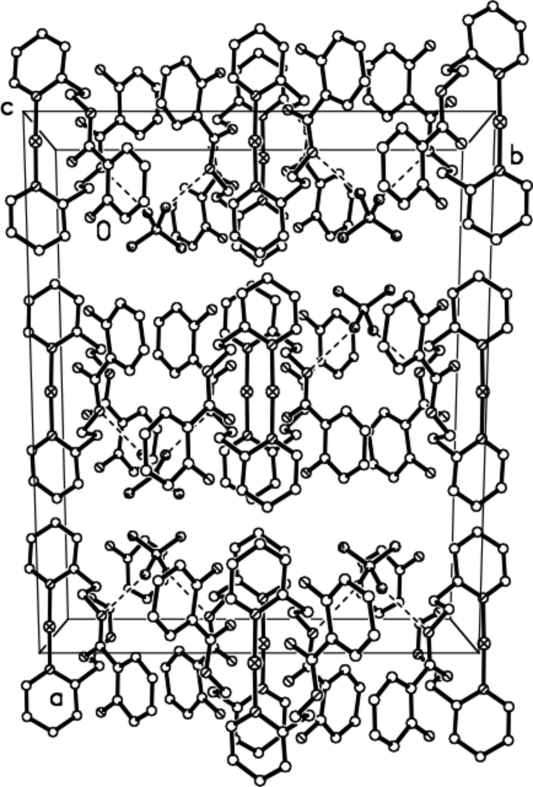
The packing of [Cu(C14H14N2O2)2]BF4, viewed down the c axis, highlighting the hydrogen bonding between complex cations and BF4- anions (dashed lines). H atoms have been omitted for clarity.

## supplementary crystallographic information

### Comment

Our laboratory has synthesized copper(I) and copper(II) complexes of pyridylmethylamide ligands that display different coordination modes depending on whether or not the amide group is protonated (Wang, *et al.* 2009, and references therein). The synthesis of copper(I) complexes with these ligands is limited to the neutral form of the ligand, where the amide group is not deprotonated (Yang, *et al.* 2007, and references therein). Attempts to synthesize copper(I) species in the presence of base results in disproportionation of the copper(I) to copper(0) and copper(II). The title complex was synthesized with the phenol-substituted ligand 2-hydroxy-*N*-(2-(2-pyridyl)ethyl)benzamide. Copper(II) complexes with 2-hydroxy-*N*-(2-(2-pyridyl)ethyl)benzamide are either mononuclear, when synthesized in the absence of base ([Cu(C_14_H_14_N_2_O_2_)_2_Cl_2_]), or a tetracopper cluster, in the presence of base ([Cu_4_(C_14_H_12_N_2_O_2_)_4_]) (Wang, *et al.* 2009). The ligand in the tetracopper cluster is a dianion with the amide NH and the phenol OH both deprotonated. The title complex differs from both copper(II) complexes in that the ligand only coordinates *via* the pyridyl N atom.

### Experimental

2-hydroxy-*N*-(2-(2-pyridyl)ethyl)benzamide was synthesized using a previously reported procedure (Wang, *et al.* 2009). The title complex, [Cu(C_14_H_14_N_2_O_2_)_2_]BF_4_, was synthesized using the following procedure: A solution of [Cu(CH_3_CN)_4_]BF_4_ (0.0778 g, 0.250 mmol) in CH_3_CN was added to a solution of 2-hydroxy-*N*-(2-(2-pyridyl)ethyl)benzamide (0.121 g, 0.500 mmol) in CH_3_CN. The resulting light yellow solution was filtered, and vapor diffusion of diethyl ether produced light yellow crystals of the title complex (0.114 g, 72% yield).

### Refinement

Hydrogen atoms bonded to C were  geometrically positioned  and refined by a riding model.  Hydrogen atom displacement parameters were set to 1.2 (1.5 for methyl) times the displacement parameters of the bonded atoms. The coordinates of the H atoms bonded to N and O were refined with U(H)=1.2U_eq_(N,O). The twin law was (1 0 0.928/ 0 -1 0/ 0 0 -1). The contribution of the minor twin domain refined to  0.437 (4).

### Crystal data

**Table:**

[Cu(C_14_H_14_N_2_O_2_)_2_]BF_4_	*F*(000) = 2608
*M**_r_* = 634.89	*D*_x_ = 1.573 Mg m^−^^3^
Monoclinic, *C*2/*c*	Mo *K*α radiation, λ = 0.71073 Å
*a* = 21.943 (5) Å	Cell parameters from 6701 reflections
*b* = 17.586 (4) Å	θ = 2.3–28.3°
*c* = 14.607 (2) Å	µ = 0.89 mm^−^^1^
β = 107.988 (8)°	*T* = 100 K
*V* = 5361.2 (19) Å^3^	Block, colorless
*Z* = 8	0.40 × 0.30 × 0.12 mm

### Data collection

**Table:**

Bruker APEX CCD diffractometer	10344 independent reflections
Radiation source: fine-focus sealed tube	8548 reflections with *I* > 2σ(*I*)
graphite	*R*_int_ = 0.0000
ω scans	θ_max_ = 26.0°, θ_min_ = 1.5°
Absorption correction: multi-scan (TWINABS; Bruker, 2001)	*h* = −27→25
*T*_min_ = 0.714, *T*_max_ = 0.905	*k* = 0→21
10344 measured reflections	*l* = 0→18

### Refinement

**Table:**

Refinement on *F*^2^	Primary atom site location: structure-invariant direct methods
Least-squares matrix: full	Secondary atom site location: difference Fourier map
*R*[*F*^2^ > 2σ(*F*^2^)] = 0.034	Hydrogen site location: inferred from neighbouring sites
*wR*(*F*^2^) = 0.096	H atoms treated by a mixture of independent and constrained refinement
*S* = 1.00	*w* = 1/[σ^2^(*F*_o_^2^) + (0.058*P*)^2^] where *P* = (*F*_o_^2^ + 2*F*_c_^2^)/3
10344 reflections	(Δ/σ)_max_ < 0.001
393 parameters	Δρ_max_ = 0.54 e Å^−^^3^
4 restraints	Δρ_min_ = −0.38 e Å^−^^3^

### Special details

**Table:**

Experimental. The selected crystal was twinned by a 2-fold rotation about an axis perpendicular to c. The ratio of the twin components was refined to 0.4123 (6).
Geometry. All e.s.d.'s (except the e.s.d. in the dihedral angle between two l.s. planes) are estimated using the full covariance matrix. The cell e.s.d.'s are taken into account individually in the estimation of e.s.d.'s in distances, angles and torsion angles; correlations between e.s.d.'s in cell parameters are only used when they are defined by crystal symmetry. An approximate (isotropic) treatment of cell e.s.d.'s is used for estimating e.s.d.'s involving l.s. planes.
Refinement. Restraints on the N—H and O—H distances were required.

### Fractional atomic coordinates and isotropic or equivalent isotropic displacement parameters (Å^2^)

**Table:**

	*x*	*y*	*z*	*U*_iso_*/*U*_eq_	
Cu1A	0.0000	0.030574 (19)	0.7500	0.01887 (10)	
N1A	0.09032 (7)	0.02912 (8)	0.79796 (11)	0.0148 (3)	
C2A	0.12104 (9)	0.08497 (11)	0.85712 (14)	0.0184 (4)	
H2A	0.0974	0.1287	0.8637	0.022*	
C3A	0.18505 (9)	0.08182 (12)	0.90856 (14)	0.0214 (5)	
H3A	0.2051	0.1224	0.9498	0.026*	
C4A	0.21963 (10)	0.01848 (12)	0.89906 (14)	0.0220 (5)	
H4A	0.2638	0.0143	0.9344	0.026*	
C5A	0.18878 (9)	−0.03911 (11)	0.83694 (14)	0.0188 (4)	
H5A	0.2120	−0.0828	0.8287	0.023*	
C6A	0.12434 (9)	−0.03273 (11)	0.78714 (13)	0.0155 (4)	
C7A	0.08947 (9)	−0.09154 (11)	0.71586 (13)	0.0181 (4)	
H7A1	0.0476	−0.1025	0.7253	0.022*	
H7A2	0.1147	−0.1392	0.7265	0.022*	
C8A	0.07893 (10)	−0.06360 (12)	0.61306 (14)	0.0223 (5)	
H8A1	0.0594	−0.0123	0.6060	0.027*	
H8A2	0.1210	−0.0591	0.6017	0.027*	
N9A	0.03805 (8)	−0.11339 (10)	0.54052 (13)	0.0205 (4)	
H9A	0.0537 (11)	−0.1414 (13)	0.5197 (16)	0.025*	
C10A	−0.02547 (10)	−0.11003 (11)	0.51659 (14)	0.0185 (4)	
O11A	−0.05148 (7)	−0.06621 (8)	0.56110 (10)	0.0255 (3)	
C12A	−0.06483 (9)	−0.15652 (11)	0.43500 (14)	0.0176 (4)	
C13A	−0.03803 (10)	−0.20269 (11)	0.37943 (14)	0.0201 (4)	
H13A	0.0072	−0.2050	0.3944	0.024*	
C14A	−0.07586 (10)	−0.24480 (12)	0.30352 (14)	0.0217 (5)	
H14A	−0.0567	−0.2753	0.2662	0.026*	
C15A	−0.14213 (10)	−0.24242 (12)	0.28176 (15)	0.0255 (5)	
H15A	−0.1683	−0.2718	0.2299	0.031*	
C16A	−0.16987 (10)	−0.19767 (12)	0.33520 (15)	0.0257 (5)	
H16A	−0.2152	−0.1961	0.3198	0.031*	
C17A	−0.13206 (10)	−0.15475 (11)	0.41143 (14)	0.0217 (5)	
O18A	−0.16259 (7)	−0.11138 (9)	0.46038 (11)	0.0311 (4)	
H18A	−0.1339 (9)	−0.0881 (13)	0.5010 (15)	0.047*	
Cu1B	0.5000	0.010247 (19)	0.7500	0.02082 (11)	
N1B	0.40965 (8)	0.00810 (9)	0.71312 (11)	0.0159 (3)	
C2B	0.37826 (10)	0.06141 (12)	0.74773 (14)	0.0216 (5)	
H2B	0.4020	0.1032	0.7823	0.026*	
C3B	0.31395 (10)	0.05821 (12)	0.73547 (15)	0.0259 (5)	
H3B	0.2937	0.0967	0.7614	0.031*	
C4B	0.27912 (10)	−0.00173 (13)	0.68487 (15)	0.0270 (5)	
H4B	0.2343	−0.0050	0.6744	0.032*	
C5B	0.31055 (9)	−0.05727 (12)	0.64944 (14)	0.0223 (5)	
H5B	0.2873	−0.0994	0.6151	0.027*	
C6B	0.37568 (9)	−0.05145 (11)	0.66406 (13)	0.0167 (4)	
C7B	0.41130 (9)	−0.10775 (11)	0.62256 (14)	0.0184 (4)	
H7B1	0.3877	−0.1566	0.6103	0.022*	
H7B2	0.4542	−0.1172	0.6691	0.022*	
C8B	0.41838 (10)	−0.07684 (11)	0.52831 (14)	0.0206 (5)	
H8B1	0.3757	−0.0758	0.4790	0.025*	
H8B2	0.4343	−0.0239	0.5387	0.025*	
N9B	0.46169 (8)	−0.12145 (9)	0.49240 (12)	0.0193 (4)	
H9B	0.4468 (10)	−0.1543 (10)	0.4524 (12)	0.023*	
C10B	0.52477 (9)	−0.11100 (10)	0.52525 (14)	0.0175 (4)	
O11B	0.54836 (7)	−0.06623 (8)	0.59362 (10)	0.0230 (3)	
C12B	0.56650 (9)	−0.15131 (10)	0.47788 (14)	0.0174 (4)	
C13B	0.54194 (10)	−0.19890 (11)	0.39787 (14)	0.0198 (4)	
H13B	0.4970	−0.2065	0.3730	0.024*	
C14B	0.58145 (11)	−0.23465 (11)	0.35483 (15)	0.0233 (5)	
H14B	0.5639	−0.2666	0.3007	0.028*	
C15B	0.64757 (11)	−0.22376 (12)	0.39108 (16)	0.0259 (5)	
H15B	0.6751	−0.2488	0.3617	0.031*	
C16B	0.67318 (10)	−0.17715 (12)	0.46890 (15)	0.0253 (5)	
H16B	0.7182	−0.1695	0.4926	0.030*	
C17B	0.63318 (10)	−0.14095 (11)	0.51318 (14)	0.0206 (4)	
O18B	0.66125 (7)	−0.09581 (9)	0.58966 (11)	0.0270 (3)	
H18B	0.6323 (8)	−0.0777 (13)	0.6054 (16)	0.032*	
B1	0.16533 (11)	0.24811 (14)	0.09405 (19)	0.0238 (5)	
F1	0.12698 (6)	0.22361 (6)	0.00247 (9)	0.0272 (3)	
F2	0.12345 (6)	0.27278 (6)	0.14410 (8)	0.0265 (3)	
F3	0.20174 (6)	0.18853 (8)	0.14239 (10)	0.0391 (3)	
F4	0.20245 (6)	0.30833 (8)	0.08330 (10)	0.0416 (4)	

### Atomic displacement parameters (Å^2^)

**Table:**

	*U*^11^	*U*^22^	*U*^33^	*U*^12^	*U*^13^	*U*^23^
Cu1A	0.01145 (18)	0.01777 (19)	0.0243 (2)	0.000	0.00099 (14)	0.000
N1A	0.0137 (8)	0.0140 (8)	0.0160 (9)	−0.0013 (6)	0.0036 (7)	0.0018 (7)
C2A	0.0203 (10)	0.0181 (10)	0.0186 (11)	−0.0015 (8)	0.0087 (8)	−0.0006 (8)
C3A	0.0189 (11)	0.0256 (11)	0.0194 (11)	−0.0056 (9)	0.0056 (9)	−0.0044 (9)
C4A	0.0132 (11)	0.0309 (12)	0.0207 (11)	−0.0022 (9)	0.0036 (8)	−0.0005 (9)
C5A	0.0171 (10)	0.0219 (10)	0.0188 (11)	0.0023 (8)	0.0077 (9)	0.0012 (8)
C6A	0.0184 (10)	0.0159 (10)	0.0133 (10)	−0.0004 (8)	0.0067 (8)	0.0031 (8)
C7A	0.0189 (10)	0.0163 (10)	0.0191 (11)	−0.0016 (8)	0.0058 (8)	−0.0007 (8)
C8A	0.0278 (12)	0.0209 (11)	0.0172 (11)	−0.0062 (9)	0.0054 (9)	−0.0016 (8)
N9A	0.0240 (10)	0.0188 (9)	0.0187 (9)	−0.0008 (7)	0.0066 (8)	−0.0055 (7)
C10A	0.0257 (11)	0.0134 (10)	0.0157 (10)	0.0025 (8)	0.0054 (8)	0.0041 (8)
O11A	0.0313 (9)	0.0235 (8)	0.0213 (8)	0.0071 (7)	0.0073 (7)	−0.0050 (6)
C12A	0.0225 (11)	0.0154 (10)	0.0149 (10)	0.0015 (8)	0.0059 (8)	0.0015 (8)
C13A	0.0203 (11)	0.0201 (10)	0.0201 (11)	−0.0011 (8)	0.0065 (9)	0.0000 (9)
C14A	0.0260 (12)	0.0208 (11)	0.0182 (11)	−0.0001 (9)	0.0068 (9)	−0.0043 (9)
C15A	0.0274 (13)	0.0240 (11)	0.0215 (12)	−0.0047 (9)	0.0022 (9)	−0.0015 (9)
C16A	0.0181 (11)	0.0301 (12)	0.0265 (12)	0.0009 (9)	0.0034 (9)	0.0032 (10)
C17A	0.0248 (11)	0.0219 (11)	0.0192 (11)	0.0052 (9)	0.0079 (9)	0.0039 (9)
O18A	0.0249 (9)	0.0375 (10)	0.0304 (9)	0.0099 (7)	0.0080 (7)	−0.0067 (7)
Cu1B	0.01183 (19)	0.02000 (19)	0.0276 (2)	0.000	0.00163 (15)	0.000
N1B	0.0136 (9)	0.0171 (8)	0.0158 (8)	0.0025 (6)	0.0027 (7)	0.0025 (7)
C2B	0.0251 (12)	0.0208 (11)	0.0165 (11)	0.0045 (9)	0.0030 (9)	0.0015 (9)
C3B	0.0268 (12)	0.0306 (12)	0.0226 (12)	0.0121 (10)	0.0109 (10)	0.0049 (10)
C4B	0.0156 (12)	0.0419 (14)	0.0249 (12)	0.0038 (9)	0.0086 (9)	0.0100 (11)
C5B	0.0182 (11)	0.0272 (11)	0.0194 (11)	−0.0043 (9)	0.0027 (9)	0.0051 (9)
C6B	0.0188 (10)	0.0182 (10)	0.0125 (10)	0.0000 (8)	0.0038 (8)	0.0050 (8)
C7B	0.0201 (11)	0.0167 (10)	0.0185 (11)	−0.0011 (8)	0.0058 (8)	0.0015 (8)
C8B	0.0244 (11)	0.0194 (10)	0.0199 (11)	0.0057 (9)	0.0097 (9)	0.0024 (8)
N9B	0.0221 (9)	0.0173 (9)	0.0190 (9)	0.0003 (7)	0.0073 (7)	−0.0035 (7)
C10B	0.0238 (11)	0.0129 (9)	0.0163 (10)	−0.0031 (8)	0.0069 (8)	0.0035 (8)
O11B	0.0286 (8)	0.0196 (7)	0.0223 (8)	−0.0059 (6)	0.0101 (6)	−0.0058 (6)
C12B	0.0207 (11)	0.0150 (10)	0.0176 (10)	−0.0002 (8)	0.0078 (8)	0.0027 (8)
C13B	0.0185 (10)	0.0199 (11)	0.0203 (11)	−0.0004 (8)	0.0049 (9)	0.0009 (9)
C14B	0.0279 (12)	0.0228 (11)	0.0192 (11)	0.0012 (9)	0.0072 (9)	−0.0025 (9)
C15B	0.0276 (12)	0.0292 (12)	0.0249 (12)	0.0076 (9)	0.0141 (10)	0.0046 (9)
C16B	0.0188 (11)	0.0303 (12)	0.0266 (12)	0.0000 (9)	0.0066 (9)	0.0061 (10)
C17B	0.0246 (11)	0.0199 (10)	0.0167 (10)	−0.0035 (9)	0.0055 (9)	0.0026 (8)
O18B	0.0227 (8)	0.0306 (9)	0.0264 (8)	−0.0066 (7)	0.0059 (7)	−0.0073 (7)
B1	0.0174 (12)	0.0237 (12)	0.0298 (14)	−0.0005 (10)	0.0064 (10)	−0.0023 (11)
F1	0.0297 (7)	0.0263 (7)	0.0266 (7)	−0.0038 (5)	0.0101 (6)	−0.0054 (5)
F2	0.0290 (7)	0.0260 (7)	0.0259 (7)	0.0064 (5)	0.0106 (6)	−0.0002 (5)
F3	0.0264 (7)	0.0396 (8)	0.0484 (9)	0.0149 (6)	0.0074 (6)	0.0076 (7)
F4	0.0320 (8)	0.0387 (8)	0.0550 (9)	−0.0173 (6)	0.0147 (7)	−0.0033 (7)

### Geometric parameters (Å, °)

**Table:**

Cu1A—N1A	1.8872 (16)	N1B—C2B	1.350 (2)
Cu1A—N1A^i^	1.8872 (16)	N1B—C6B	1.355 (2)
N1A—C2A	1.344 (2)	C2B—C3B	1.368 (3)
N1A—C6A	1.356 (2)	C2B—H2B	0.9500
C2A—C3A	1.374 (3)	C3B—C4B	1.376 (3)
C2A—H2A	0.9500	C3B—H3B	0.9500
C3A—C4A	1.379 (3)	C4B—C5B	1.385 (3)
C3A—H3A	0.9500	C4B—H4B	0.9500
C4A—C5A	1.389 (3)	C5B—C6B	1.382 (3)
C4A—H4A	0.9500	C5B—H5B	0.9500
C5A—C6A	1.381 (3)	C6B—C7B	1.500 (3)
C5A—H5A	0.9500	C7B—C8B	1.532 (3)
C6A—C7A	1.499 (3)	C7B—H7B1	0.9900
C7A—C8A	1.528 (3)	C7B—H7B2	0.9900
C7A—H7A1	0.9900	C8B—N9B	1.450 (2)
C7A—H7A2	0.9900	C8B—H8B1	0.9900
C8A—N9A	1.452 (2)	C8B—H8B2	0.9900
C8A—H8A1	0.9900	N9B—C10B	1.330 (3)
C8A—H8A2	0.9900	N9B—H9B	0.815 (11)
N9A—C10A	1.330 (3)	C10B—O11B	1.251 (2)
N9A—H9A	0.72 (2)	C10B—C12B	1.487 (3)
C10A—O11A	1.254 (2)	C12B—C13B	1.403 (3)
C10A—C12A	1.483 (3)	C12B—C17B	1.405 (3)
C12A—C13A	1.399 (3)	C13B—C14B	1.371 (3)
C12A—C17A	1.408 (3)	C13B—H13B	0.9500
C13A—C14A	1.378 (3)	C14B—C15B	1.396 (3)
C13A—H13A	0.9500	C14B—H14B	0.9500
C14A—C15A	1.390 (3)	C15B—C16B	1.373 (3)
C14A—H14A	0.9500	C15B—H15B	0.9500
C15A—C16A	1.376 (3)	C16B—C17B	1.395 (3)
C15A—H15A	0.9500	C16B—H16B	0.9500
C16A—C17A	1.388 (3)	C17B—O18B	1.354 (2)
C16A—H16A	0.9500	O18B—H18B	0.805 (11)
C17A—O18A	1.356 (2)	B1—F3	1.373 (3)
O18A—H18A	0.829 (12)	B1—F4	1.374 (3)
Cu1B—N1B	1.8874 (17)	B1—F2	1.408 (3)
Cu1B—N1B^ii^	1.8874 (17)	B1—F1	1.410 (3)
			
N1A—Cu1A—N1A^i^	178.45 (9)	C6B—N1B—Cu1B	121.61 (13)
C2A—N1A—C6A	118.45 (17)	N1B—C2B—C3B	123.2 (2)
C2A—N1A—Cu1A	119.31 (13)	N1B—C2B—H2B	118.4
C6A—N1A—Cu1A	121.37 (13)	C3B—C2B—H2B	118.4
N1A—C2A—C3A	123.17 (18)	C2B—C3B—C4B	118.8 (2)
N1A—C2A—H2A	118.4	C2B—C3B—H3B	120.6
C3A—C2A—H2A	118.4	C4B—C3B—H3B	120.6
C2A—C3A—C4A	118.61 (19)	C3B—C4B—C5B	118.9 (2)
C2A—C3A—H3A	120.7	C3B—C4B—H4B	120.6
C4A—C3A—H3A	120.7	C5B—C4B—H4B	120.6
C3A—C4A—C5A	118.86 (19)	C6B—C5B—C4B	120.0 (2)
C3A—C4A—H4A	120.6	C6B—C5B—H5B	120.0
C5A—C4A—H4A	120.6	C4B—C5B—H5B	120.0
C6A—C5A—C4A	119.89 (19)	N1B—C6B—C5B	120.84 (18)
C6A—C5A—H5A	120.1	N1B—C6B—C7B	116.97 (17)
C4A—C5A—H5A	120.1	C5B—C6B—C7B	122.11 (18)
N1A—C6A—C5A	121.00 (18)	C6B—C7B—C8B	109.81 (16)
N1A—C6A—C7A	116.95 (17)	C6B—C7B—H7B1	109.7
C5A—C6A—C7A	122.00 (17)	C8B—C7B—H7B1	109.7
C6A—C7A—C8A	110.51 (16)	C6B—C7B—H7B2	109.7
C6A—C7A—H7A1	109.5	C8B—C7B—H7B2	109.7
C8A—C7A—H7A1	109.5	H7B1—C7B—H7B2	108.2
C6A—C7A—H7A2	109.5	N9B—C8B—C7B	113.13 (16)
C8A—C7A—H7A2	109.5	N9B—C8B—H8B1	109.0
H7A1—C7A—H7A2	108.1	C7B—C8B—H8B1	109.0
N9A—C8A—C7A	113.27 (17)	N9B—C8B—H8B2	109.0
N9A—C8A—H8A1	108.9	C7B—C8B—H8B2	109.0
C7A—C8A—H8A1	108.9	H8B1—C8B—H8B2	107.8
N9A—C8A—H8A2	108.9	C10B—N9B—C8B	121.34 (17)
C7A—C8A—H8A2	108.9	C10B—N9B—H9B	119.9 (15)
H8A1—C8A—H8A2	107.7	C8B—N9B—H9B	118.7 (15)
C10A—N9A—C8A	121.77 (18)	O11B—C10B—N9B	120.18 (19)
C10A—N9A—H9A	121.2 (19)	O11B—C10B—C12B	120.59 (18)
C8A—N9A—H9A	117.0 (19)	N9B—C10B—C12B	119.21 (17)
O11A—C10A—N9A	119.95 (18)	C13B—C12B—C17B	118.15 (18)
O11A—C10A—C12A	120.69 (18)	C13B—C12B—C10B	122.59 (18)
N9A—C10A—C12A	119.33 (18)	C17B—C12B—C10B	119.25 (18)
C13A—C12A—C17A	117.85 (18)	C14B—C13B—C12B	121.41 (19)
C13A—C12A—C10A	122.77 (18)	C14B—C13B—H13B	119.3
C17A—C12A—C10A	119.37 (17)	C12B—C13B—H13B	119.3
C14A—C13A—C12A	121.46 (19)	C13B—C14B—C15B	119.59 (19)
C14A—C13A—H13A	119.3	C13B—C14B—H14B	120.2
C12A—C13A—H13A	119.3	C15B—C14B—H14B	120.2
C13A—C14A—C15A	119.71 (19)	C16B—C15B—C14B	120.5 (2)
C13A—C14A—H14A	120.1	C16B—C15B—H15B	119.7
C15A—C14A—H14A	120.1	C14B—C15B—H15B	119.7
C16A—C15A—C14A	120.2 (2)	C15B—C16B—C17B	120.1 (2)
C16A—C15A—H15A	119.9	C15B—C16B—H16B	120.0
C14A—C15A—H15A	119.9	C17B—C16B—H16B	120.0
C15A—C16A—C17A	120.4 (2)	O18B—C17B—C16B	117.34 (18)
C15A—C16A—H16A	119.8	O18B—C17B—C12B	122.40 (18)
C17A—C16A—H16A	119.8	C16B—C17B—C12B	120.26 (19)
O18A—C17A—C16A	117.29 (19)	C17B—O18B—H18B	105.6 (17)
O18A—C17A—C12A	122.33 (18)	F3—B1—F4	112.05 (19)
C16A—C17A—C12A	120.38 (19)	F3—B1—F2	109.72 (19)
C17A—O18A—H18A	105.4 (18)	F4—B1—F2	109.07 (18)
N1B—Cu1B—N1B^ii^	177.71 (9)	F3—B1—F1	109.67 (19)
C2B—N1B—C6B	118.27 (17)	F4—B1—F1	109.17 (19)
C2B—N1B—Cu1B	119.44 (14)	F2—B1—F1	107.02 (17)
			
C6A—N1A—C2A—C3A	−1.2 (3)	C6B—N1B—C2B—C3B	−0.1 (3)
Cu1A—N1A—C2A—C3A	168.21 (15)	Cu1B—N1B—C2B—C3B	−170.83 (15)
N1A—C2A—C3A—C4A	0.1 (3)	N1B—C2B—C3B—C4B	−0.5 (3)
C2A—C3A—C4A—C5A	0.9 (3)	C2B—C3B—C4B—C5B	1.0 (3)
C3A—C4A—C5A—C6A	−1.0 (3)	C3B—C4B—C5B—C6B	−0.8 (3)
C2A—N1A—C6A—C5A	1.2 (3)	C2B—N1B—C6B—C5B	0.3 (3)
Cu1A—N1A—C6A—C5A	−168.01 (14)	Cu1B—N1B—C6B—C5B	170.80 (14)
C2A—N1A—C6A—C7A	−176.21 (17)	C2B—N1B—C6B—C7B	177.21 (16)
Cu1A—N1A—C6A—C7A	14.6 (2)	Cu1B—N1B—C6B—C7B	−12.3 (2)
C4A—C5A—C6A—N1A	−0.1 (3)	C4B—C5B—C6B—N1B	0.2 (3)
C4A—C5A—C6A—C7A	177.15 (18)	C4B—C5B—C6B—C7B	−176.57 (18)
N1A—C6A—C7A—C8A	74.4 (2)	N1B—C6B—C7B—C8B	−80.1 (2)
C5A—C6A—C7A—C8A	−102.9 (2)	C5B—C6B—C7B—C8B	96.8 (2)
C6A—C7A—C8A—N9A	−172.26 (17)	C6B—C7B—C8B—N9B	169.84 (16)
C7A—C8A—N9A—C10A	82.1 (2)	C7B—C8B—N9B—C10B	−82.9 (2)
C8A—N9A—C10A—O11A	−4.3 (3)	C8B—N9B—C10B—O11B	5.6 (3)
C8A—N9A—C10A—C12A	173.60 (17)	C8B—N9B—C10B—C12B	−172.57 (16)
O11A—C10A—C12A—C13A	175.99 (18)	O11B—C10B—C12B—C13B	−176.59 (18)
N9A—C10A—C12A—C13A	−1.9 (3)	N9B—C10B—C12B—C13B	1.6 (3)
O11A—C10A—C12A—C17A	−4.0 (3)	O11B—C10B—C12B—C17B	2.5 (3)
N9A—C10A—C12A—C17A	178.11 (18)	N9B—C10B—C12B—C17B	−179.32 (18)
C17A—C12A—C13A—C14A	0.5 (3)	C17B—C12B—C13B—C14B	0.1 (3)
C10A—C12A—C13A—C14A	−179.47 (19)	C10B—C12B—C13B—C14B	179.18 (18)
C12A—C13A—C14A—C15A	−0.7 (3)	C12B—C13B—C14B—C15B	0.0 (3)
C13A—C14A—C15A—C16A	0.6 (3)	C13B—C14B—C15B—C16B	−0.5 (3)
C14A—C15A—C16A—C17A	−0.2 (3)	C14B—C15B—C16B—C17B	0.8 (3)
C15A—C16A—C17A—O18A	179.36 (19)	C15B—C16B—C17B—O18B	179.90 (18)
C15A—C16A—C17A—C12A	0.0 (3)	C15B—C16B—C17B—C12B	−0.7 (3)
C13A—C12A—C17A—O18A	−179.44 (18)	C13B—C12B—C17B—O18B	179.63 (18)
C10A—C12A—C17A—O18A	0.5 (3)	C10B—C12B—C17B—O18B	0.5 (3)
C13A—C12A—C17A—C16A	−0.1 (3)	C13B—C12B—C17B—C16B	0.2 (3)
C10A—C12A—C17A—C16A	179.85 (18)	C10B—C12B—C17B—C16B	−178.91 (18)

Symmetry codes: (i) −*x*, *y*, −*z*+3/2; (ii) −*x*+1, *y*, −*z*+3/2.

### Hydrogen-bond geometry (Å, °)

**Table:**

*D*—H···*A*	*D*—H	H···*A*	*D*···*A*	*D*—H···*A*
N9A—H9A···F1^iii^	0.72 (2)	2.23 (2)	2.923 (2)	161 (2)
O18A—H18A···O11A	0.83 (1)	1.79 (2)	2.554 (2)	152 (3)
N9B—H9B···F2^iv^	0.82 (1)	2.16 (1)	2.937 (2)	159 (2)
O18B—H18B···O11B	0.81 (1)	1.81 (2)	2.549 (2)	153 (2)

Symmetry codes: (iii) *x*, −*y*, *z*+1/2; (iv) −*x*+1/2, *y*−1/2, −*z*+1/2.
